# Decrease in the Ratio proBDNF/BDNF in the Urine of Aging Female Patients with OAB

**DOI:** 10.3390/metabo13060723

**Published:** 2023-06-03

**Authors:** Claudia Covarrubias, Philippe G Cammisotto, Samer Shamout, Lysanne Campeau

**Affiliations:** 1 Lady Davis Institute, McGill University, Montreal, QC H3A 0G4, Canada; claudia.covarrubias@mail.mcgill.ca (C.C.);; 2 Urology Department, Jewish General Hospital, Montreal, QC H3T 1E2, Canada

**Keywords:** BDNF, proBDNF, urine, overactive bladder, proteases, MMP-9, microRNAs

## Abstract

Imbalance in the levels of neurotrophins, growth factors crucial in the development, function, and survival of neurons is commonly observed in many pathological states. Concentrations of brain-derived neurotrophic factor (BDNF) and its precursor (proBDNF) were measured in the urine of a cohort of aging female patients with overactive bladder disease (OAB). When reported to creatinine, levels were similar between OAB patients and healthy controls. However, the ratio proBDNF/BDNF was significantly decreased in the OAB group. Receiver operating characteristic (ROC) curve analysis of the ratio proBDNF/BDNF displayed a good diagnostic value for OAB (AUC = 0.729). Clinical questionnaires of symptom severity (OABSS and IIQ-7) were negatively correlated with this ratio. On the other hand, microRNAs (miRNA) involved in proBDNF gene translation were expressed at comparable levels between groups. However, urinary enzymatic activity of matrix metalloproteinase-9 (MMP-9), the enzyme that cleaves proBDNF into BDNF, was increased in OAB compared to controls. Levels of miR-491-5p, the main miRNA that downregulates MMP-9 synthesis, were greatly decreased in urine from OAB patients. These results suggest that the ratio proBDNF/BDNF could be useful in the phenotyping of OAB in an aging population, and the difference could originate from enhanced MMP-9 enzymatic activity rather than translational control.

## 1. Introduction

Overactive bladder (OAB) is defined by the International Continence Society (ICS) as a syndrome of urinary urgency, frequency and nocturia, in the absence of urinary tract infection (UTI) or other obvious pathology. Urgency, the hallmark symptom of OAB, is described as the “complaint of a sudden, compelling desire to pass urine which is difficult to defer” [1,2] and can accompany or precede incontinence (urgency urinary incontinence), characterized as involuntary leakage of urine. OAB can be sub-categorized as dry (OAB-dry), meaning without incontinence, or wet, with incontinence (OAB-wet) [1].

The overall prevalence of OAB is estimated at 10–12% within the adult population, with increasing rates with aging [3,4,5]. Additionally, the current literature describes women having a higher prevalence of OAB-wet when compared to their male counterpart [4,5,6]. Through population-based studies, the increase in the prevalence of OAB in women has been linked to several factors including advanced age, menopause, marital status, increased body mass index (BMI), and high parity rates [3,6,7]. Altogether, the symptoms of OAB have detrimental effects on psychosocial functioning and overall quality of life (QoL) [4]. There is no clear etiology of non-neurogenic OAB; rather, it is likely a manifestation of several contributing factors. Treatment modalities primarily provide symptomatic relief as there is currently a lack of curative treatment [2]. Behavioral therapy is recommended as first-line treatment, followed by second-line treatment with oral pharmacotherapy, which includes antimuscarinics and β3-adrenoceptor agonists [8]. Refractory OAB or intolerable medication side effects can warrant third-line therapeutics such as intravesical OnabotulinumtoxinA, sacral neuromodulation, or peripheral tibial nerve stimulation [2,8].

Over the past several years, various hypotheses have been proposed to explain the pathophysiological mechanisms and identify clinical subtypes of OAB [9]. It is well documented that bladder function, such as urine storage and voiding, is controlled by the peripheral and central nervous systems, relying on the interconnection between the autonomic and somatic nervous systems [10]. Neurotrophins are a class of growth factors that were originally found in the nervous system where they promote growth and survival of neurons, directly regulating nerve ending activity [11]. In particular, brain-derived neurotrophic factor (BDNF) is synthesized by many cell types outside of the nervous system (e.g., megakaryocytes, neurons, endothelial cells) including bladder smooth muscle cells and constitutes the most abundant neurotrophin in the human body [12]. Mature BDNF originates from a precursor (proBDNF) after proteolytic cleavage [13]. Mature BDNF binds preferentially to high-affinity receptor tropomyosin receptor kinase B (TrkB), to promote neuro-regeneration and axonal growth. On the other hand, proBDNF binds the proinflammatory/apoptotic p75 neurotrophin receptor (p75^NTR^), triggering inflammation and apoptosis [14,15]. The ratio between mature BDNF and proBDNF determine the balance between survival and apoptotic cellular pathways and depends upon the expression of several intracellular and extracellular metalloproteinases (MMPs) and convertases [13]. In particular, the metalloproteinase MMP-9 promotes extracellular proBDNF conversion into mature BDNF (mBDNF) [16]. Both neurotrophins have been proposed to be markers of OAB [17].

OAB is a clinical diagnosis where severity is quantified with patient reported outcomes [2]. Identifying the different phenotypes of OAB according to their underlying causative factors could possibly highlight therapeutic targets. Given the aforementioned, it is necessary to develop a non-invasive, objective, valid and reproducible test for the diagnosis, phenotyping and therapeutic targeting of OAB. Herein, we propose the use of urinary neurotrophins and associated metalloproteinases as biomarkers for OAB in an aging female population. The objectives of our study were to: (1) measure the ratio proBDNF/BDNF to improve the phenotyping of OAB and (2) determine changes in the concentrations of microRNAs and proteins associated with these neurotrophins to understand the molecular mechanisms of the underlying pathophysiology.

## 2. Materials and Methods

### 2.1. Patient Profiles

Participants in the OAB group were women aged between 50 and 80 years-old (*n* = 20) who were diagnosed with OAB (with or without treatment) and were recruited at the urology department of the Jewish General Hospital, Montreal, Canada (see flow diagram, Supplementary Materials Figure S1). Their symptoms include urinary frequency and urgency, with or without urge incontinence, for at least 3 months. They also were required to withhold all OAB treatments (anticholinergic and beta-3 agonist) for at least 3 weeks before the sample collection. A routine negative screening urine culture to exclude urinary tract infection (UTI) was also performed. The control subjects (20) group were normal volunteers or patients attending the same clinic within the same age group (50–80 years old) who had no urinary symptoms, no current or prior use of OAB medications, and a negative urine test for any infection. Exclusion criteria were as follows: established diabetes mellitus, history of malignancies or pelvic radiotherapy, pelvic organ prolapse, urinary tract infection, neurogenic lower urinary tract dysfunction, and hepatic or renal impairment (creatinine clearance <70 mL/min). All patients were interviewed in person. Informed written consent was provided by all patients. This study was approved by the Medical-Biomedical Research Ethics Committee (REC) of the Integrated Health and Social Services University Network for West-Central Montreal (IRB: 2016-328, 15-022, approved on 20 June 2017).

A sample size calculation was previously carried out using the original project estimation based on the Human Metabolome Data Base. Urine succinate level was used as a reference based on previous metabolomic studies performed [18,19,20]. Differences between control and disease conditions were 3.4 μmol/mmol creatinine (with normal urine succinate at 5.6 and abnormal of 9.0 μmol/mmol creatinine), with a standard deviation (sd) of 3.8, a study power at 80% and significance at 0.05.

### 2.2. Demographic and Clinical Differences

Complete medical history, physical examination, screening urinalysis, 1-day voiding diary and validated symptom questionnaires were carried out on every participant. Voiding dairies allowed us to estimate: 24 h, daytime and nighttime frequencies, total 24 h voided volume, nocturnal voided volume, mean voided volume per micturition, and maximum voided volume. All participants completed the Overactive Bladder Symptom Score (OABSS), the International Consultation on Incontinence Questionnaire-Short Form (ICIQ-SF), and the Incontinence Impact Questionnaire (IIQ-7) [21,22]. Few patients only recorded their urgency and leakage episode in the voiding diary and hence they were not statistically tested due to insufficient data. Fasting glucose and insulin levels were measured to calculate Homeostatic Model Assessment of Insulin resistance (HOMA-IR) as an index for insulin resistance. Significant insulin resistance was determined by values above 2.9.

### 2.3. Collection and Preparation of Urine Samples

Midstream early morning urine samples were gathered by patients in two sterile plastic containers. One was kept at 4 °C for bacterial culture. The other container was kept at −20 °C. Dietary restrictions were not requested during urine collection. Upon reception at the hospital, samples were thawed, aliquoted and stored at −80 °C. Laboratory staff was blinded to which samples were OAB or controls.

### 2.4. MiRNAs Isolation from Urine Samples

Urine aliquots were kept on ice then centrifuged at 10,000 rpm for 20 min to remove particles (cells and cellular debris). Subsequently, a urine microRNA (miRNA) purification kit (Norgen Biotek protocol Corp, Thorold, ON, Canada) was used to isolate miRNAs from supernatants, according to manufacturer’s protocol. These columns isolate total miRNAs (cell-free and vesicular/exosomal ones), as well as small nuclear RNAs [23]. No DNAse or RNAse treatments were required. Assessment of contamination by DNA was carried out by using the Nanodrop system for single strand DNA and double strand DNA: no significant amount of DNA was found. A RNA contamination test was not carried out as the Norgen isolation kit specifically isolates miRNA and small RNAs, the latter being used as reference to standardize miRNA measurements. Quantification of nucleic acid was carried out on a nanodrop system. The purity of the nucleic acid (RNA) (A260/A280) was close to 2 for all samples. RNA integrity was also assessed by the Nanodrop system.

### 2.5. MicroRNA Poly-Adenylation and Synthesis of cDNA

A polymerase tailing kit from Lucigen (Middleton, WI, USA) was used to add poly(A)(adenine) tails to mature miRNAs. In short, purified RNAs were incubated with adenosine triphosphate (ATP, 1 mM) and *Escherichia coli* (*E. coli*) poly(A) polymerase (200 U/mL) for 30 min at 37 °C. The reaction was ended by incubating samples for 5 min at 95 °C. Subsequently, complementary DNA (cDNA) synthesis was performed using a custom-made stem loop primer containing a poly-T(thymidine) tail (Integrated DNA Technologies (IDT, Coralville, IA, USA)). Reverse transcriptase kit (OneScript cDNA synthesis kit) from abm (Richmond, BC, Canada) was used according to the manufacture’s protocols, with the following incubation settings: Reverse transcription (RT) for 30 min, 50 °C for 50 min and 85 °C for 5 min. Samples were held at 4 °C, transferred to −20 °C for long term storage. Reference gene small nuclear RNA U6 (snU6) was amplified with a specific primer [23]. Total cDNA obtained was quantified using a Nanodrop system.

### 2.6. Quantitative PCR (qPCR)

Primers were purchased from Integrated DNA Technologies (IDT, Coralville, IA, USA). Universal primer complementary to the stem loop primer was used together with forward primers specific for each miRNA of interest (See Supplementary Materials Tables S1 and S2). The reference gene snU6 was detected with its own set of forward and reverse primers. Quantitative PCRs were carried out on a Sensifast Probe Low-ROX (low carboxyrhodamine) kit containing Synergy Brands SYBR-green, on an Applied Bioscience 7500 Fast Real-Time PCR, under the following conditions: 95 °C 10 min, 45 cycles of 95 °C 15 s and 60 °C 35 s, always followed by melt curve analysis. Samples were analyzed in triplicates. Primers were tested for specificity and efficiency (90–110%). Relative miRNA expressions were analyzed using the 2^−ΔΔCT^ method [24].

### 2.7. Enzyme-Linked Immunosorbent Assay (ELISA) and Enzymatic Kits

ELISA kits for BDNF, proBDNF and p75^NTR^ extracellular domain (p75^ECD^) were purchased from Biosensis (Thebarton, Australia), those for sortilin and cortisol were from Abcam (Cambridge, MA, USA). Enzymatic kits were from the following providers: matrix metalloproteinase-3 (MMP-3) (AAT Bioquest, CA, USA), matrix metalloproteinase-7 (MMP-7), matrix metalloproteinase-9 (MMP-9) (Quickzyme, Leidan, The Netherlands), and plasmin (Sigma-Aldrich, Oakville, ON, Canada).

### 2.8. Statistics

Comparisons between groups were achieved by Student *t*-test (demographics, voiding diary, serum data and questionnaires) or Mann–Whitney test (not normally distributed). Significance was set at *p* < 0.05. One-way analysis of covariance (ANCOVAs) for confounders (age, homeostatic model assessment for insulin resistance (HOMA-IR) and estimated glomerular filtration rate (eGFR)) were performed to compare differences between control and OAB cohorts. Spearman’s correlation was used to analyze urinary parameters, questionnaires’ scores, and voiding diary parameters. Receiver operating characteristic (ROC) was computed to determine sensitivity and specificity. IBM SPSS Statistics ver. 23.0 (IBM Co., Armonk, NY, USA) was used for all statistics.

## 3. Results

### 3.1. Subject Characteristics

Of the 52 total female participants enrolled in this study, only 40 participants successfully completed the study protocol and are included in the analysis. The mean age for the OAB group was higher than the control group (68.9 ± 11.38 vs. 56.25 ± 5.22 years in controls, *p* < 0.001) (Table 1). There was no significant difference in the body mass index (BMI), demographics, or vital signs between both groups. The OAB group was found to have higher HOMA-IR level (3.11± 1.18 vs. 2.13± 1.03 in controls, *p* = 0.020), prevalence of metabolic syndrome, and hypertension when compared to the control group. Additionally, OAB symptom severity as reflected on the voiding diary and questionnaires’ scores, were significantly higher in the OAB group (Table 1). Patients from the OAB group presented higher HOMA-IR level and higher prevalence of metabolic syndrome (40%) and hypertension (65%) compared to patients from the control group (20% of metabolic syndrome and hypertension, each). On the other hand, voiding diary and total questionnaires’ scores, which reflect OAB symptom severity and its impact on quality of life, were as well significantly higher in the OAB group (Table 1).

### 3.2. Biochemical Urinalysis

**Figure 1:**
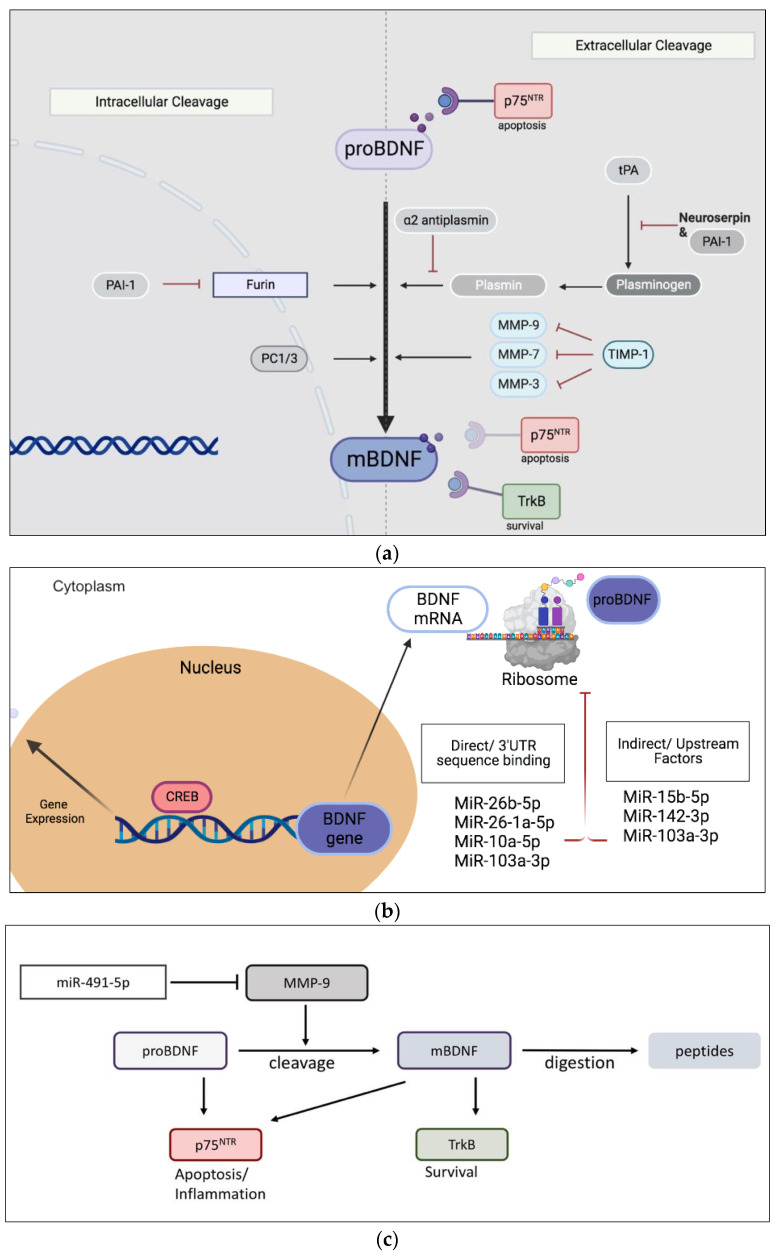
(**a**) Schematic representation of proteolytic pathways converting precursor proBDNF to its mature form mBDNF. (**b**) General illustration of BDNF ribosomal synthesis. Several miRNAs elicit direct and indirect inhibition of BDNF mRNA expression. Binding of BDNF and proBDNF to TrkB rand p75^NTR^ receptors, respectively. (**c**) MiR-491-5p inhibition of MMP-9 implicated in the metabolism of BDNF. MiR, microRNA; TrkB, tyrosine kinase receptor B; PAI-1, plasminogen activator inhibitor-1; PC1/3, proprotein convertase 1/3; tPA, tissue plasminogen activator; TIMP-1, tissue inhibitor of metalloproteinase-1; mBDNF, mature brain-derived neurotrophic factor; MMP, matrix metalloproteinases; p75^NTR^, p75 neurotrophin receptor; UTR, untranslated regions; CREB, cAMP response element-binding protein. Figure (**a**,**b**) were created using BioRender.

All variables tested summarized in Figure 1 were corrected to creatinine levels.

**Figure 2:**
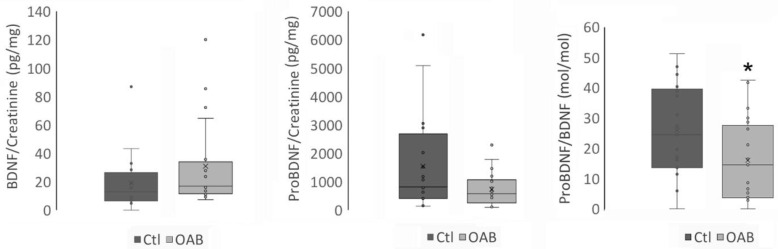
BDNF and proBDNF levels in urine samples from control (Ctl) and OAB patients (OAB). Levels of BDNF and proBDNF were measured in parallel and normalized to creatinine. The ratio proBDNF/BDNF is also represented. (Ctl *n* = 20, OAB *n* = 20). Non-parametric Whitney test was carried out. * *p* < 0.05.

There were no differences in single standing BDNF/creatinine levels in the urine of controls versus OAB patients (Table 2 and Figure 2), while proBDNF/creatinine measures were lower in the OAB population, yet not statistically significant. The ratio of proBDNF/BDNF was significantly lower in the OAB group (*p =* 0.023) (Table 2 and Figure 2).

**Figure 3:**
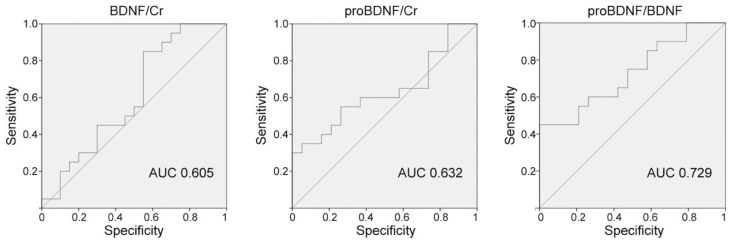
Receiver Operating Characteristics (ROC) curve for BDNF/creatinine and proBDNF/creatinine levels in urine samples. Area under curve (AUC) was computed for BDNF/creatinine (BDNF/Cr) (pg/mg creatinine), proBDNF/creatinine (proBDNF/Cr) (pg/mg creatinine), and the ratio proBDNF/BDNF (mol/mol). The highest AUC was found for the ratio proBDNF/BDNF.

Receiver operating characteristic (ROC) for proBDNF/BDNF demonstrated high sensitivity for OAB diagnosis (AUC = 0.729) compared to each neurotrophin taken separately (Figure 3).

**Figure 4:**
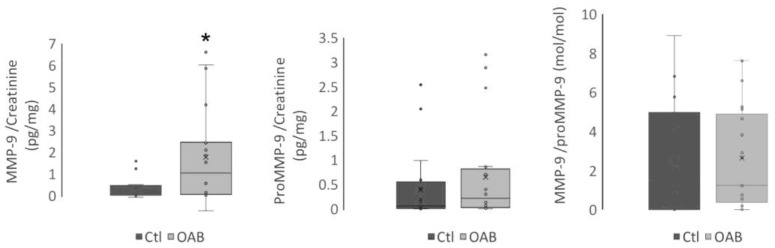
Matrix metalloproteinase-9 (MMP-9) and its precursor proMMP-9 enzymatic activity in urine samples from control (Ctl) and OAB patients (OAB). Activity of MMP-9 and total amount of proMMP-9 were measured and normalized to creatinine. The ratio MMP-9/proMMP-9 were also plotted. (Ctl *n* = 20, OAB *n* = 20). Non-parametric Whitney test was carried out. * *p* < 0.05.

Additionally, enzymatic activity of MMP-9, one of the main enzymes converting proBDNF to BDNF, was higher in the OAB group: 0.325 ± 0.124 vs. 1.802 ± 0.481 ng/mg creatinine in control and OAB group, respectively, *p* = 0.035 (Table 2). MMP-9 and its precursor proMMP-9 enzymatic activities were also plotted to further assess this trend (Figure 4). The ratio imbalance suggested an increased conversion of proBDNF to BDNF due to an enhanced activity of MMP-9.

We further adjusted the urinary levels of BDNF, MMP-9, and their precursor molecules with metabolic confounders of age, HOMA-IR index, and estimated kidney function level (eGFR). The urinary levels of proBDNF/BDNF ratio in the OAB group remained lower in a statistically significant fashion after adjusting for HOMA-IR and eGFR separately (*p <* 0.05) (Table 3).

Subsequently, correlation between BDNF, proBDNF and clinical questionnaires in the total cohort showed that the OABSS score had a significant negative correlation with standalone proBDNF levels (*p* <0.05). The proBDNF/BDNF levels also negatively correlated with OABSS and IIQ-7 (*p* <0.05) but did not show this trend with the ICIQ-SF questionnaire (Table 4).

To provide insights in the regulation of proBDNF synthesis, we measured diverse urinary factors and microRNAs known to control the translation of proBDNF mRNA (Table 5). MiR-26b-5p, miR-26a-5p, miR-10a-5p, and miR-103a-3p that bind the 3′UTR sequence of proBDNF mRNA were not expressed differently between control and OAB patients [25,26]. Levels of other miRNAs (miR-15b-5p, miR-142-3p and miR-103a-3p) that control proBDNF expression through downstream or upstream pathways were also not different [25,27,28]. On the other hand, concentration of miR-491-5p, which negatively controls MMP-9 expression, was significantly decreased in the OAB group: 0.122 (Q1, Q3: 0.0221, 0.392) vs. 0.533 (Q1,Q3: 0.302, 1.643) in the control group, *p* < 0.05 (Figure 1c) [29]. Another factor associated with BDNF synthesis, cortisol, was not significantly different (Table 5) [30]. Finally, level of soluble extracellular domain of receptor p75 (p75^ECD^) resulting from the cleavage of membrane-bound receptor p75^NTR^ was increased in the OAB group while concentrations of the p75^NTR^ co-receptor sortilin were similar [31,32]. Adjustments of miR-491-5p and p75^ECD^ data to clinical questionnaires were still highly correlated (Supplementary Materials Table S3).

## 4. Discussion

The present study examined the diagnostic and phenotyping value of the urinary ratio proBDNF over mature BDNF for OAB. Levels of microRNAs and proteins related to proBDNF synthesis and proteolysis of mature BDNF were determined along with the concentration of receptors sortilin and p75^ECD^, which are involved in proinflammatory processes after binding proBDNF.

A recent metanalysis gathered evidence on urinary mature BDNF/creatinine as a potential biomarker for OAB [33]. The concentrations of BDNF measured in the present study are in the range of those reported in previous publications (between 4.7 to 859 pg/mg creatinine for controls and between 4.0 to 1627 pg/mg creatinine for OAB patients). Differences in proBDNF/BDNF ratios between OAB and control group were still found after correcting for HOMA-IR and eGFR confounders, suggesting that they do not contribute to the proBDNF/BDNF ratio imbalance found in OAB. Unlike previously published studies on OAB patient cohorts [34,35], we did not observe single standing difference of BDNF/Cr levels between both groups [36,37]. An explanation for this can be that BDNF is the most abundant of the neurotrophins in the human body [38]. Likewise, studies have shown that the aging process has no significant impact on BDNF concentrations, which is a characteristic that is not shared by nerve growth factor (NGF) [39]. The activation of MMP-9 proteolysis of proBDNF should lead to a subsequent increase in BDNF, which we did not observe. This could be explained by other pathways targeting the downregulation of BDNF activity. In vitro, we observed that nitric oxide (NO), which has been shown to be increased during OAB, decreases BDNF release by bladder smooth muscle cells (SMCs) in culture (unpublished observations). The interaction between NO and BDNF could be explained by impaired release of BDNF by cells caused by NO induced decreased in calcium influx in smooth muscle [40].

We have reported a higher proNGF/NGF ratio in this OAB population compared to controls [30]. Compensatory mechanism between inter-neurotrophin level variations has been described in Sprague Dawley rats [41]. Therefore, the inverse finding of a lower proBDNF/BDNF ratio here described could be explained by a compensatory MMP-9 proteolysis to increase BDNF levels.

According to recent reports, little is known regarding the role of BDNF in bladder function, due to the limited number of studies, the low number of patients involved and the diversity of detection kits used [33]. In particular, the ELISA kits used were not tested for their specificity to distinguish between BDNF and proBDNF, given the similarity in sequence present in both forms. We confirmed the specificity of our kits (unpublished materials), as previously conducted for NGF and proNGF [17]. Nevertheless, we can extrapolate the results on BDNF and proBDNF thanks to their specific signaling pathways. It is well known that neurotrophins and their precursors trigger different pathways by binding specific membrane-bound receptors associated with cell survival and growth or to inflammation and apoptosis [42]. The relative amount of pro- and mature neurotrophins could have more clinical value than each taken separately [42].

On the other hand, the ratio proBDNF/BDNF presents a similar sensitivity than the proNGF/NGF in detecting patients with OAB (ROC analysis 0.729 vs. 0.735, respectively) [30]. Our findings are in accordance with previous studies reporting a diagnostic value of BDNF/creatinine between 0.67 and 0.95, which is considered as a ‘fair’ performance [31].

Levels of miRNAs involved in the control of proBDNF translation were unchanged, confirming our previous report that the balance of proNGF/NGF appears to be controlled at the level of protease activity rather than at a transcriptional step [17]. In accordance, enzymatic activity of MMP-9 converting proBDNF to BDNF was enhanced in OAB. In vitro experiment on bladder primary cell cultures using the gene editing enzyme Crispr-Cas9 to partially delete MMP-9 gene led to massive accumulation of proBDNF in the culture medium (unpublished observation), highlighting the crucial role of MMP-9 in proBDNF proteolysis. Indeed, the upregulated MMP-9 activity observed in the OAB group can be caused by the associated lower levels of miR-491-5p, which negatively controls MMP-9 expression by direct binding of a 3′-UTR sequence present on MMP-9 mRNA. Within the field of oncology, the tumor suppressor miR-491-5p expression has been reported to inhibit important metastatic pathways [29]. Exploring the role of microRNAs in OAB pathophysiology could uncover potential therapeutic targets.

Finally, we found a statistically significant increase in the extracellular portion of p75^NTR^ receptor in urine of OAB patients. The p75^ECD^ is downregulated in neurological disease and has been suggested to possess neuroprotective properties [31]. A recent report showed an increase in plasma p75^ECD^ after 4 weeks treatment of diabetic type 1 mice with an antagonist of p75^NTR^ [32]. In the present study, a relatively lower level of proBDNF binding to p75^NTR^ would decrease its activation and lead to an increase in p75^ECD^. The physiologic implications of this phenomenon remain to be elucidated.

The outcome of this pilot study provides empirical evidence to further support the utility of neurotrophins as biomarkers for OAB and simultaneously presents the underlying pathophysiological mechanisms of OAB symptomology. ProBDNF/BDNF ratio was found to have a significantly negative correlation with the scores of OAB symptom questionnaires (OABSS, IIQ-7). This suggests that the dysregulated levels of the balance of the biological isoforms of BDNF, proinflammatory/apoptotic proBDNF, and neuroprotective BDNF contribute more to the pathogenesis of OAB than either one alone. For instance, research within the domain of neuropsychology has suggested that proBDNF/BDNF ratio may be more an indicator of cognitive change than proBDNF and BDNF levels alone [43]. Our data demonstrate that the ratio of proBDNF/BDNF and MMP-9 activity complement each other in the presence of OAB, and that this can be directly linked to microRNA activity and common metabolic comorbidities highly prevalent in the aging population (e.g., systemic arterial hypertension, dyslipidemia, and impaired glucose tolerance). Additionally, our previous study found an increased urinary level of NO and prostaglandin E2 (PGE2), two co-activators of MMP-9, which further highlights the importance of MMP-9 activity in the pathophysiology of OAB through the dysregulation of BDNF isoforms [17,44,45].

This pilot study is subject to several limitations, including a small sample size. As we did not consider patients with non-idiopathic OAB or male patients, our results cannot be generalized to other patient groups outside of our inclusion criteria. Other clinical limitations associated with this study are further discussed in our previous publications [17,18]. Future larger clinical studies should assess a larger sample of subjects with varying disease evolution and severity, with follow-up and response to treatments. This would allow a better understanding of the clinical value of proBDNF and associated metabolites as prognostic, diagnostic, and early markers of OAB development, and particularly help phenotype patients according to different etiologies. This in turn could allow us to further validate results that use proBDNF metabolism as a tool for tailored treatment protocols.

## 5. Conclusions

In conclusion, the present report suggests that the ratio proBDNF/BDNF could help improve diagnosis and phenotyping of OAB in female patients in a context of metabolic syndrome. More research is required to comprehend the role of these neurotrophins in the development of the pathology. It also confirms our previous report on NGF suggesting that neurotrophins are regulated at the levels of proteolytic enzymes converting pro-form to mature form rather than the translational levels. Finally, it strengthens the fact that the ratio between precursor and mature neurotrophins has more diagnostic value than isolated values of mature neurotrophins alone.

## Figures and Tables

**Table 1:** Characteristics of control and OAB groups: Demographic, serum, symptom questionnaires, and urine analysis data.

	CTR	OAB Group	*p* Value
Demographic and serum analysis:			
Age (years)	56.25 (5.22)	68.9 (11.38)	<0.001
BMI (kg/m^2^)	29.75 (7.65)	28.82 (5.45)	ns
eGFR (mL/min/1.73 m^2^)	98.5 (14.52)	76 (19.78)	<0.001
HOMA-IR	2.13 (1.03)	3.11 (1.18)	0.020
Total Cholesterol/HDL	3.50 (1.18)	3.23 (0.81)	ns
Questionnaires’ scores:			
OABSS (0–28)	7.3 (3.56)	17.45 (4.45)	<0.001
ICIQ--SF (0–22)	3.26 (3.98)	8.05 (3.83)	<0.001
IIQ-7 (0–100)	2.4 (5.2)	28.9 (23.2)	<0.001
Voiding diary parameters:			
24 h frequency	9.15 (2.28)	11.4 (3.03)	0.012
Daytime frequency	8.5 (2.04)	9.5 (2.09)	ns
Night frequency	0.65 (0.81)	1.9 (1.71)	0.005
24 h voiding volume (mL)	2705 (2346.02)	1859.6 (865.37)	ns
Night voiding volume (mL)	495.25 (253.88)	449.75 (270.77)	ns
Mean voided volume (mL)	322.25 (311.1)	167.36 (75.2)	0.037
Maximum voided volume (mL)	480.75 (193.44)	327.25 (126.7)	0.005

Data are presented as mean (Standard Deviation) for variables compared with independent *t*-test. Statistically significant differences are reported with *p* value. (ns) non-significant. Abbreviations: Body Mass Index (BMI), estimated Glomerular Filtration Rate (eGFR), Homeostatic Model Assessment for Insulin Resistance (HOMA-IR), high density lipoproteins (HDL), Overactive Bladder Symptom Score (OABSS), International Consultation on Incontinence Questionnaire-Short Form (ICIQ-SF), and Incontinence Impact Questionnaire (IIQ-7).

**Table 2:** Urinary BDNF, proBDNF, MMP-9, and proMMP-9 levels compared between control and OAB groups without considering confounders.

	Ctl Group	OAB Group	*p* Value
BDNF (pg/mg creat)	19.67 ± 4.25	30.92 ± 6.84	0.265
proBDNF (pg/mg creat)	1543.5 ± 381.7	733.1 ± 131.8	0.231
proBDNF/BDNF	27.24 ± 3.09	16.89 ± 3.02	0.023
MMP-9 (ng/mg creat)	0.325 ± 0.124	1.802 ± 0.481	0.035
proMMP-9 (ng/mg creat)	0.391 ± 0.162	0.646 ± 0.216	0.301
MMP-9/pro-MMP-9	2.401 ± 0.60	3.446 ± 0.61	0.251

Data are presented as mean ± SEM for variables compared with the non-parametric Mann–Whitney test. Significant differences are reported (*p* < 0.05).

**Table 3:** Urinary BDNF, MMP-9, and their precursors compared between control (CTR) and OAB groups considering confounders.

	Confounders	CTR	OAB Group	*p* Value
BDNF (pg/mg creat)	Age	27.51 (20.63–34.39)	26.87 (19.99–33.76)	0.952
	HOMA-IR	20.78 (13.95–27.6)	27.66 (20.59–34.74)	0.509
	eGFR	22.97 (16.1–29.9)	31.41 (13.99–48.84)	0.431
proBDNF (pg/mg creat)	Age	1528 (1202–1853)	749 (424–1074)	0.133
	HOMA-IR	1753 (1390–2117)	729 (352–1105)	0.072
	eGFR	1488 (1169–1807)	789 (470–1108)	0.162
proBDNF/BDNF (mol/mol)	Age	24.81 (21.44–28.18)	19.31 (15.94–22.68)	0.302
	HOMA-IR	29.45 (26.08–32.82)	18.89 (15.4–22.38)	0.047
	eGFR	27.59 (24.18–31.01)	16.53 (13.11–19.95)	0.042
MMP-9 (ng/mg creat)	Age	0.530 (0.082–0.978)	1.645 (1.225–2.065)	0.111
	HOMA-IR	0.581 (0.108–1.054)	1.677 (1.204–2.150)	0.128
	eGFR	0.445 (0.014–0.876)	1.722 (1.316–2.128)	0.054
proMMP-9 (ng/mg creat)	Age	0.583 (0.374–0.792)	0.455 (0.246–0.664)	0.697
	HOMA-IR	0.387 (0.170–0.604)	0.539 (0.314–0.764)	0.645
	eGFR	0.331 (0.117–0.545)	0.707 (0.376–1.038)	0.262
MMP-9/proMMP-9	Age	2.419 (1.682–3.156)	3.431 (2.740–4.122)	0.367
	HOMA-IR	2.727 (1.889–3.565)	3.764 (2.957–4.571)	0.405
	eGFR	3.010 (2.342–3.678)	2.900 (2.271–3.529)	0.913

Data are presented as estimated marginal mean (95% CI) for variables compared with ANCOVA. Statistically significant differences are considered at *p* < 0.05. Abbreviations: Body Mass Index (BMI), estimated Glomerular Filtration Rate (eGFR), Homeostatic Model Assessment for Insulin Resistance (HOMA-IR).

**Table 4:** Correlation between BDNF, proBDNF, and symptom questionnaires in the total cohort.

		Correlation	*p* Value
BDNF (pg/mg creat)	OABSS	0.035	0.828
	ICIQ-SF	0.165	0.314
	IIQ-7	0.144	0.377
proBDNF (pg/mg creat)	OABSS	−0.336	0.034
	ICIQ-SF	−0.176	0.285
	IIQ-7	−0.267	0.096
proBDNF/BDNF (mol/mol)	OABSS	−0.392	0.012
	ICIQ-SF	−0.290	0.073
	IIQ-7	−0.391	0.013
MMP-9 (ng/mg creat)	OABSS	0.259	0.117
	ICIQ-SF	0.307	0.065
	IIQ-7	0.207	0.212

BDNF, proBDNF and the ratio proBDNF/BDNF were correlated to three questionnaires, OABSS, ICIQ-SF and IIQ-7. The *p* value for Spearman correlation was considered statistically significant for *p* < 0.05. Abbreviations: Overactive Bladder Symptom Score (OABSS), International Consultation on Incontinence Questionnaire-Short Form (ICIQ-SF), and Incontinence Impact Questionnaire (IIQ-7).

**Table 5:** Urinary factors involved in proBDNF synthesis and proteolysis in the control and OAB groups.

	Ctl	OAB	***p*** Value
miR-26b-5p	0.891 (0.423, 1.864)	1.865 (0.0830, 2.842)	0.99
miR-26a-5p	0.808 (0.495, 1.911)	1.634 (0.0852, 2.911)	0.989
miR-10a-5p	0.884 (0.420, 3.489)	0.344 (0.101, 1.083)	0.108
miR-103a-3p	1.200 (0.500, 2.156)	0.592 (0.0570, 1.105)	0.102
miR-15b-5p	0.825 (0.494, 3.076)	1.504 (0.307, 4.070)	0.841
miR-142-3p	0.438 (0.0728, 1.511)	0.449 (0.267, 1.930)	0.369
miR-202-3p	1.300 (0.318, 2.750)	0.257 (0.146, 2.531)	0.211
miR-124-5p	0.939 (0.382, 4.121)	1.356 (0.259, 2.234)	0.813
miR-152-5p	1.362 (1.166, 3.902)	0.721 (0.298, 5.568)	0.792
miR-491-5p	0.533 (0.302, 1.643)	0.122 (0.0221, 0.392)	0.008
MMP-7 (ng/mg creat)	0.232 (0.140, 0.455)	0.443 (0.228, 1.02)	0.079
MMP-3 (mU/mg creat)	0.0147 (0.00584, 0.0262)	0.0104 (0.00038, 0.235)	0.583
Plasmin (ng/mg creat)	18.1 (14.57, 30.90)	27.65 (15.65, 38.40)	0.512
Cortisol (ng/mg creat)	38.18 (29.72, 53.74)	35.29 (17.89, 56.70)	0.529
p75^ECD^ (ng/mg creat)	2.471 (2.149, 2.855)	2.851 (2.623, 3.593)	0.035
Sortilin (pg/mg creat)	1710 (737, 2984)	1752 (1286, 3266)	0.398

MicroRNAs binding the 3′UTR part of the BDNF gene (miR-26-50, miR-26-1a-5p, miR-10a-5p, miR-103a-3p) or involved in its indirect upstream control (miR-202-3p, miR-10a-5p, miR-15b-5p, miR-142-3p) were measured by RTqPCR and normalized to snU6. Enzymatic activities other than MMP-9 involved in the conversion of proBDNF to BDNF (MMP-3, MMP-7 and plasmin) were measured. Another factor associated with BDNF synthesis, cortisol, and membrane-bound proteins involved in proBDNF signaling (sortilin and p75^ECD^) were assessed as well. Data are presented as median (interquartile range, Q1, Q3) for variables compared with the non-parametric Mann–Whitney test. Significant differences are reported in bold (*p* < 0.05).

## Data Availability

Data are unavailable due to privacy or ethical restrictions.

## Supplementary Materials

The following supporting information can be downloaded at: https://www.mdpi.com/article/10.3390/metabo13060723/s1, Figure S1: Flow diagram underlying the study selection process; Table S1: qPCR miRNA information; Table S2: Primer sequences used for qPCR; Table S3: Correlation between miR-491-5p and p75^ECD^ with symptom questionnaires’ scores and voiding diary parameters.
