# Supplementary material for: Decrease in the Ratio proBDNF/BDNF in the Urine of Aging Female Patients with OAB
Source: Metabolites. 2023 Jun 3;13(6):723. doi: 10.3390/metabo13060723 (PMC10303400; doi:10.3390/metabo13060723)
Supplement: Supplementary file 1 [file metabolites-13-00723-s001.zip › metabolites-2407969-supplementary.pdf]

Figure S1: Flow diagram underlying the study selection process.

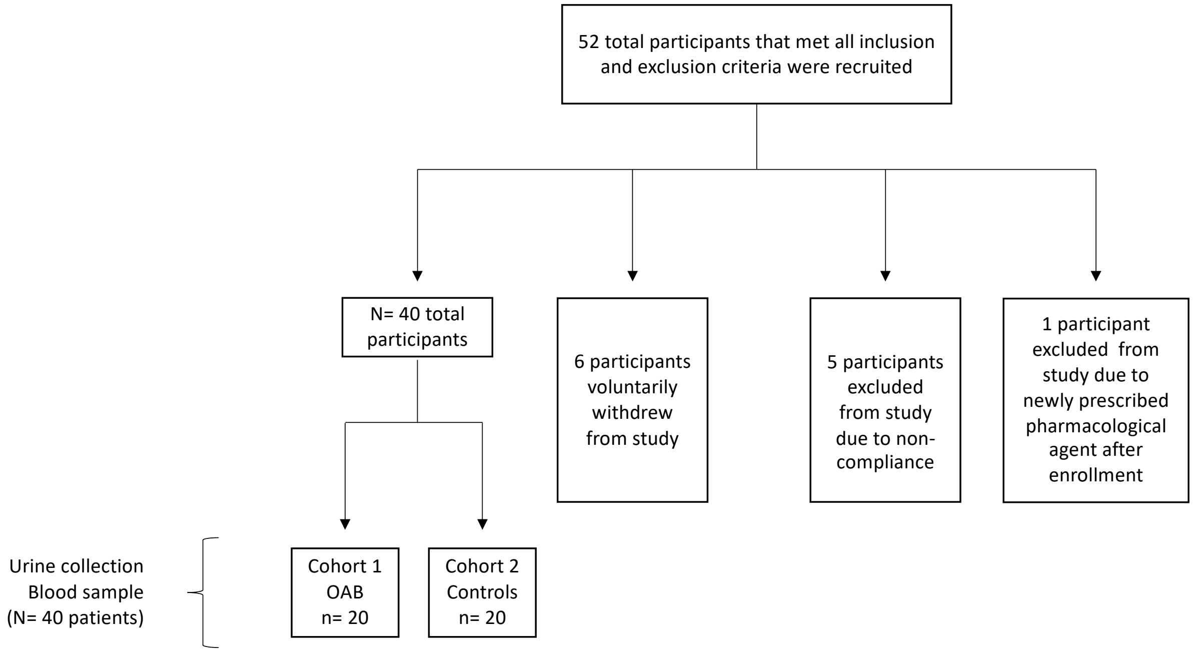

Table S1: qPCR miRNA information.

| | Sequence accession number |
|---|---|
| **miR-26b-5p** | MIMAT0000083 |
| **miR-26a-5p** | MIMAT0000082 |
| **miR-10a-5p** | MIMAT0000253 |
| **miR-491-5p** | MIMAT0002807 |
| **miR-103a-3p** | MIMAT0009196 |
| **miR-15b-5p** | MIMAT0000417 |
| **miR-142-3p** | MIMAT0000433 |
| **miR-202-3p** | MIMAT0002810 |
| **miR-124-5p** | MIMAT0004591 |
| **miR-152-5p** | MIMAT0026479 |
| **snU6** | NR 004394.1 |

Table S2: Primer sequences used for qPCR

| | primers |
|---|---|
| **miR-26b-5p** | ACACTCCAGCTGGGTTCAAGTAATTCAGG |
| **miR-26a-5p** | ACACTCCAGCTGGGTTCAAGTAATCCAGG |
| **miR-10a-5p** | ACACTCCAGCTGGGTACCCTGTAGATCCG |
| **miR-491-5p** | ACACTCCAGCTGGGAGTGGGGAACCCT |
| **miR-103a-3p** | ACACTCCAGCTGGGAGCAGCATTGTACAG |
| **miR-15b-5p** | ACACTCCAGCTGGGTAGCAGCACATCATG |
| **miR-142-3p** | ACACTCCAGCTGGGCATAAAGTAGAAAGC |
| **miR-202-3p** | ACACTCCAGCTGGGAGAGGTATAGGGCAT |
| **miR-124-5p** | ACACTCCAGCTGGGCGTGTTCACAGCGGA |
| **miR-152-5p** | ACACTCCAGCTGGGCATAAAGTAGAAAGC |
| **universal primer** | CTCACAGTACGTTGGTATCCTTGTG |
| **snU6 forward** | CTCGCTTCGGCAGCACATATACT |
| **snU6 reverse** | ACGCTTCACGAATTTGCGTGTC |

Table S3: Correlation between miR-491-5p and p75[ECD] with symptom questionnaires' scores and voiding diary parameters:

| miRNA | Variables | Correlation coefficient (r) | p value |
|---|---|---|---|
| miR-491-5p[$] | OABSS | -0.405 | **0.011** |
| | ICIQ-SF | -0.379 | **0.019** |
| | IIQ-7 | -0.494 | **0.001** |
| p75[ECD] | OABSS | -0.372 | **0.018** |
| | ICIQ-SF | -0.539 | **0.0001** |
| | IIQ-7 | -0.372 | **0.018** |

The p value for Spearman correlation, only $p < 0.05$ are reported as considered statistically significant.

[$] Data on miR-491-5p were published in Metabolites (same special issue), Cammisotto et al (2022).
